# Cardiac Rehabilitation in TAVI Patients: Safety and Benefits: A Narrative Review

**DOI:** 10.3390/medicina61040648

**Published:** 2025-04-01

**Authors:** Theodor Constantin Stamate, Cristina Andreea Adam, Radu Sebastian Gavril, Radu Ștefan Miftode, Andreea Rotundu, Ovidiu Mitu, Doina Clementina Cojocaru, Grigore Tinică, Florin Mitu

**Affiliations:** 1 Department of Medical Specialties I, “Grigore T. Popa” University of Medicine and Pharmacy, 700115 Iasi, Romania; stamate.theodor@gmail.com (T.C.S.); adam.cristina93@gmail.com (C.A.A.); radu-stefan.miftode@umfiasi.ro (R.Ș.M.); andreea.rotundu@gmail.com (A.R.); ovidiu.mitu@umfiasi.ro (O.M.); doina.cojocaru@umfiasi.ro (D.C.C.); florin.mitu@umfiasi.ro (F.M.); 2 Doctoral School, “Grigore T. Popa” University of Medicine and Pharmacy, 700115 Iasi, Romania; 3 Clinical Rehabilitation Hospital, 700661 Iasi, Romania; 4 Department of Cardiology, “St. Spiridon” Emergency County Hospital, 700111 Iasi, Romania; 5 Institute of Cardiovascular Disease “Prof. Dr. George I.M. Georgescu”, 700503 Iasi, Romania; grigoretinica@yahoo.com; 6 Department of Cardiac Surgery, Faculty of Medicine, “Grigore T. Popa” University of Medicine and Pharmacy, 700115 Iasi, Romania; 7 Romanian Academy of Medical Sciences, 030167 Bucharest, Romania; 8 Romanian Academy of Scientists, 050045 Bucharest, Romania

**Keywords:** cardiac rehabilitation, transcatheter aortic valve implantation, frailty, exercise training, aortic stenosis

## Abstract

Transcatheter aortic valve implantation (TAVI) has redefined the management of severe aortic stenosis, particularly in surgical high-risk patients. As the number of TAVI procedures increases, there is a growing need for effective post-procedural care. Cardiac rehabilitation (CR) has emerged as a critical component of treatment in these patients. The most recent update of the European recommendations highlights the importance of including post-TAVI patients in CR programs. However, the benefits of CR in this particular patient group still need to be fully understood. The objective of this narrative review is to summarize the safety and benefits of post-TAVI CR by evaluating its impact on functional capacity, frailty, muscular strength, mental health, quality of life, and long-term survival. While emerging evidence supports its safety and effectiveness in the aforementioned outcomes, gaps remain regarding the optimal rehabilitation protocols, including the timing, duration, and intensity of CR as well as its long-term cardiovascular benefits. Further research is needed to develop personalized approaches for different patient groups. This article highlights the current knowledge, identifies critical gaps, and underlines the need for tailored rehabilitation strategies to improve post-TAVI recovery and patient outcomes.

## 1. Introduction

Transcatheter aortic valve implantation (TAVI) is a percutaneous procedure designed to replace the aortic valve, which is indicated for patients with severe aortic stenosis and high surgical risk, predominantly elderly, frail, and with multiple comorbidities [1]. The latest update of the European recommendations promotes inclusion of post-TAVI patients in cardiac rehabilitation (CR) programs [2]. However, the benefits of CR in this particular patient group still need to be fully understood. This narrative review aims to provide a broad and integrative perspective of the current body of knowledge by summarizing recent research findings, highlighting key areas of consensus among studies, and identify persisting gaps that warrant further investigation.

## 2. Materials and Methods

A comprehensive literature search was conducted across the PubMed, EMBASE, and MEDLINE databases, from inception to 12 December 2024. Since TAVI is a relatively recent procedure, first introduced in 2002, all studies related to TAVI were considered for inclusion, regardless of the year of publication.

Studies were selected based on the MeSH terms outlined below: aortic valve stenosis, transcatheter aortic valve implantation, aortic valve replacement, exercise and cardiac rehabilitation. Eligible studies were required to meet the following criteria: (1) assessment of exercise-based CR in TAVI patients; (2) exercise-based CR interventions independent of supervision (supervised or unsupervised), setting (hospital-based or home-based), care setting (inpatient or outpatient), or the inclusion of educational or psychological support (with or without); (3) inclusion of meta-analyses, randomized controlled trials (RCTs), cohort studies, or observational studies; (4) written in English. Studies were eligible for inclusion if they provided outcomes measured both prior to and following the rehabilitation program. Primary endpoints were either compared within a single group (pre- and post-intervention) or between an intervention group (receiving CR) and a control group (receiving usual care). Studies that included a mixed population of SAVR and TAVI patients were eligible, provided that the TAVI subgroup was clearly defined and analyzed separately. Exclusion criteria were as follows: (1) animal or non-human studies; (2) publications in the form of abstracts, letters, editorials, expert opinions, or case reports; (3) studies lacking sufficient data or failing to meet the inclusion criteria; (4) studies involving patients who had previously participated in a defined exercise-based rehabilitation program. The authors provided a narrative synthesis of the included studies containing the name of the first author, year, country, type of study, number of TAVI patients, outcome measurements, follow-up period, and main findings.

## 3. Aortic Stenosis: Epidemiology, Prognosis, and the Evolving Role of Transcatheter Aortic Valve Implantation in Patient Management

### 3.1. Epidemiology

Aortic stenosis (AS) is the most prevalent valve disease that requires surgical intervention in both Europe and North America [1], with its degenerative form showing an exponential increase with aging. In recent decades, population aging has been a sustained demographic process. Recent studies predict a rise in the number of individuals over 60 years old from 962.3 million in 2017 to 2.08 billion in 2050 [3]. Consequently, an increase in the number of AS cases is expected. The Euro Heart Survey, a large prospective real-world study of 5001 adults from 25 European countries diagnosed with moderate-to-severe native valvular heart disease, infective endocarditis, or prior valve interventions, stated AS to be the most common native valve lesion, followed by mitral regurgitation, aortic regurgitation, and mitral stenosis [4]. As shown in other similar studies conducted in Europe and North America, the majority of AS cases are due to a degenerative mechanism (81.9%), followed by rheumatic etiology [4,5]. A meta-analysis reported a prevalence of AS of 12.4% among individuals over 75 years old, with severe forms accounting for 3.4% [6].

### 3.2. Prognosis

The natural progression of AS includes a prolonged asymptomatic phase [7]. During this period, the disease may be incidentally diagnosed during a physical examination by detecting a systolic ejection murmur at the aortic focus or through resting electrocardiographic changes, which typically prompt echocardiography for confirmation. Once symptoms such as dyspnea, angina, or syncope develop, the prognosis becomes poor [7]. The risk of sudden death increases from approximately 1% annually in asymptomatic severe AS to 10–20% in symptomatic patients [8]. Without valve replacement, symptomatic patients have a 5-year survival rate of only 50% [9]. For those with concurrent heart failure, the mortality risk rises to 50% within 2 years [10]. The worst outcomes are seen in patients with severe symptomatic AS accompanied by significant left ventricular systolic dysfunction [11].

Drug therapy has not been shown to effectively slow the progression of aortic AS [1]. Although it was hypothesized that statins could play a beneficial role, recent meta-analyses have disproven this theory [1,12]. The only proven effective treatment is valve replacement, performed either through traditional surgical methods or via transcatheter interventions.

### 3.3. TAVI—A New Alternative Since 2002

The first transcatheter aortic valve implantation procedure was performed by Cribier et al. in 2002, offering a viable alternative to traditional surgery for patients with symptomatic severe aortic stenosis who are at elevated risk for perioperative mortality [13]. Current European guidelines recommend that the decision between surgical and interventional approaches must be made by a multidisciplinary heart team comprising experts in valvular diseases including cardiovascular surgeons, cardiologists, imaging specialists, anesthesiologists, internists, and electrophysiologists [1].

Indications for TAVI are determined by both clinical and anatomical/procedural factors. Clinical factors include high surgical risk, advanced age, frailty, and a history of cardiac surgery. Anatomical factors include suitability for a transfemoral approach, the presence of post-radiotherapy sequelae, porcelain aorta, a high likelihood of patient–prosthesis mismatch (aortic valve area < 0.65 cm^2^/m^2^), severe chest deformities, scoliosis, and the presence of associated cardiac pathology requiring intervention. These conditions include septal hypertrophy requiring myomectomy, significant aortic root or ascending aorta dilation or aneurysm, severe primary mitral or tricuspid valve disease, and multivessel coronary artery disease [1].

In recent years, there has been a paradigm shift, with TAVI now being considered for patients with low surgical risk [14]. Randomized clinical trials such as PARTNER 3 and Evolut Low Risk compared transcatheter intervention with traditional aortic valve replacement surgery [15,16,17]. These studies demonstrated lower rates of mortality, stroke, and heart failure hospitalizations for TAVI. A recent meta-analysis, which included seven randomized trials, reported a reduced risk of all-cause mortality and stroke at 2 years for patients treated with the interventional approach compared with those undergoing surgery [18]. Therefore, given the dynamic nature of this patient population, there is a need to gain a deeper understanding of the role of cardiac rehabilitation in this evolving context.

## 4. Cardiac Rehabilitation in Cardiovascular Disease: Components, Indications, and Pathophysiology Implications of Exercise-Based Intervention

**Figure 1:**
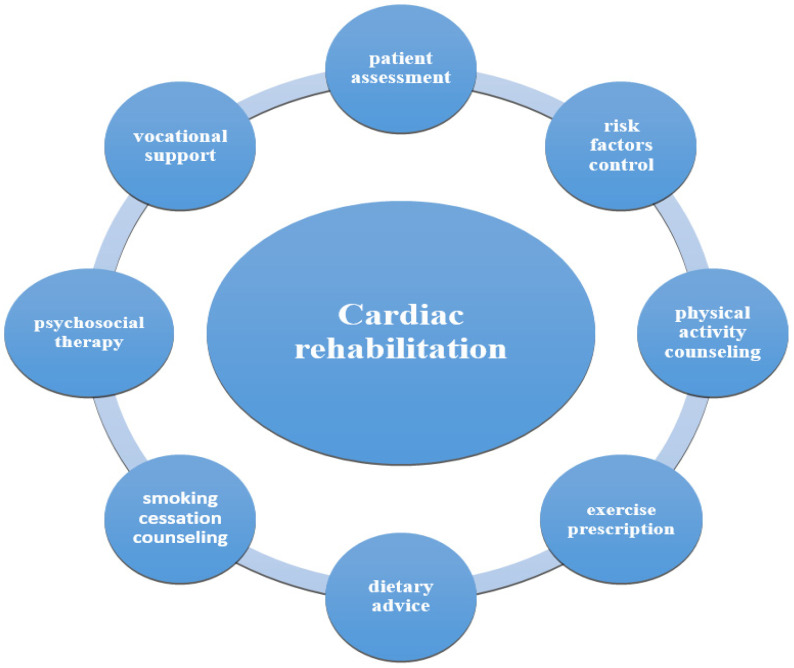
Components of cardiac rehabilitation.

Cardiac rehabilitation involves a range of key elements, including patient evaluation, risk factor identification and management, physical activity guidance, exercise plans, nutritional counseling, smoking cessation support, psychosocial therapy, and vocational assistance [2,19,20,21] (Figure 1). CR is strongly recommended for various cardiovascular conditions including angina, chronic heart failure, post-myocardial infarction, surgical or interventional revascularization procedures, heart transplantation, and valvular surgery [2].

The latest update to these recommendations was published in March 2020 [2]. The position paper of the European Association of Preventive Cardiology of the ESC recommends CR for new clinical conditions and special populations including frailty syndrome patients, those undergoing TAVI or MitraClip procedures, individuals with implanted cardiac devices or ventricular assist devices, and non-compliant patients. It aligns lipid metabolism and hypertension control targets with the latest guidelines and defines the optimal intensity for aerobic training. Resistance training and inspiratory muscle training are recommended for chronic heart failure, and the guidelines also provide a comprehensive approach for managing frail patients. Moreover, the position paper underlines the importance of evaluating and addressing psychosocial risk factors as well as vocational issues related to returning to professional activity [2].

Cardiac rehabilitation is very well studied among patients with heart failure with reduced ejection fraction, post-myocardial infarction, or after coronary revascularization [22]. In these, CR has been shown to enhance functional capacity and improve quality of life and risk factor management. Additionally, CR has led to reductions in cardiovascular mortality and hospitalization rates [23,24,25].

From a pathophysiological standpoint, the benefits of exercise-based interventions derive from multiple aspects, including the reduction in key risk factors, enhancement of lipid metabolism, improved adherence to prescribed treatments, identification and management of depression, decreased insulin resistance, better glucose regulation, and the normalization of fibrinolytic activity [23,26,27,28].

Exercise-induced improvement in endothelial function, primarily through increased shear stress, promotes the production of nitric oxide. This vasodilatory mediator improves vascular tone, reduces inflammation, and enhances blood flow. After TAVI, patients often face a compromised endothelial state due to aging and underlying heart disease, so exercise can restore endothelial health [29].

TAVI patients often experience autonomic imbalance, with sympathetic overactivity and parasympathetic withdrawal, leading to a higher heart rate and lower heart rate variability (HRV). Exercise has been shown to improve autonomic function by enhancing vagal activity, which lowers the resting heart rate and improves HRV. Additionally, regular physical activity modulates the neurohormonal system by reducing circulating catecholamine levels, decreasing the sympathetic drive, and promoting vasodilation, which helps manage blood pressure and reduce arrhythmic potential post-TAVI [30].

Furthermore, there is evidence of favorable effects on cardiac remodeling, including decreased left ventricular filling pressures and enhanced atrioventricular dynamics [27]. Regular physical activity enhances myocardial efficiency by improving cardiac output and reducing oxygen demand through improved mitochondrial function and capillary density [31]. It also reverses frailty by increasing muscle strength and endurance while combating sarcopenia through resistance training, which promotes muscle hypertrophy [32].

## 5. Post-TAVI Cardiac Rehabilitation: Safety and Benefits; State of the Art and Evidence from the Literature; and Gaps in Knowledge

Currently, most post-TAVI patients present with a vulnerable phenotype: elderly, frail, with high surgical risk and multiple comorbidities [33,34]. Therefore, safety is a primary consideration in implementing cardiac rehabilitation programs. Table 1 provides an overview of the key meta-analyses in the field, while Table 2 highlights the principal clinical trials.

### 5.1. Safety of the Cardiac Rehabilitation Program in TAVI Patients

A key finding from the referenced studies [35,37,48,49,52] is the confirmed safety of exercise-based cardiac rehabilitation programs. This conclusion is supported by a meta-analysis published by Ribeiro et al. that included 5 studies comprising 292 TAVI patients undergoing CR [35]. Results indicated that CR was safe after TAVI regardless of the type, frequency, and intensity of the rehabilitation program. Although TAVI patients were older and had greater functional impairments compared with those treated surgically, both groups tolerated the CR program similarly. Notably, none of the five studies included in the meta-analysis reported any deaths related to cardiac rehabilitation.

A randomized 8-week pilot trial with a small sample size of 27 TAVI patients was conducted to evaluate the efficacy and safety of exercise-based CR in post-TAVI care [49]. The intervention included moderate, progressively increased endurance training on a cyclo-ergometer and resistance exercises, performed 2–3 times per week for 20–45 min per session. Safety was assessed by recording adverse clinical events that resulted in either an extended interruption of the intervention (lasting more than one week) or its complete cessation. Monitoring included renal function (via creatinine levels and glomerular filtration rate) and prosthesis function post-intervention. Any adverse effects that happened during or within 12 h following an exercise session were categorized as training-related. The results demonstrated that physical training and exercise-based evaluations, such as cardiopulmonary exercise testing (CPET) and one-repetition maximum testing, were completed safely. Two participants discontinued the study due to incidents occurring on non-exercise days and more than 24 h after the last training session. No evidence of prosthesis impairment was observed during the study. Only one patient in the usual care group exhibited an aggravation of paravalvular regurgitation from mild to moderate. Furthermore, participating in CR had no effect on NT-proBNP levels and renal function, supporting the safety of exercise-based CR in this population.

A recent prospective randomized controlled trial evaluating the impact of moderate-intensity continuous training (MICT) in 66 post-TAVI patients reported 2 adverse clinical events throughout the trial period [40]. These included one cardiac death in the MICT group that was not related to exercise, while in the control group, there was one death from cardiogenic shock and aggravated heart failure. Importantly, both events were not associated with the training program and occurred on resting days.

A study by Zanettini et al. evaluating the effects of a cardiac rehabilitation program exclusively in TAVI patients highlighted the high frailty, functional impairment, and dependency within this patient population, alongside a significant risk of clinical complications [53]. Approximately 1/3 of the participants experienced complications; however, only 3.3% (2 patients) required transfer to an acute care hospital, and no deaths occurred during the program. Overall, outcomes were positive, with notable improvements in functional status, quality of life, and autonomy. While significant adverse events during inpatient CR have been reported, no adverse events were directly linked to the delivery of the intervention. Instead, these occurrences underline the important role of CR in the early detection and management of clinical complications.

Participation in an exercise-based CR program does not lead to prosthetic dysfunction or worsening valvular regurgitation after the intervention [35,40,46,53].

This evidence strongly highlights the safety of exercise-based CR, including both aerobic and resistance training, even in patients with significant disability, frailty, or multiple comorbidities following TAVI.

### 5.2. Benefits of the Cardiac Rehabilitation Program in TAVI Patients

The diversity of study designs in the literature on CR in TAVI patients reflects a range of research objectives and patient populations. While randomized controlled trials provide the highest-quality evidence, other designs, such as observational studies and retrospective analyses, still play an essential role in assessing the broad applicability and long-term benefits of CR. Studies comparing TAVR patients with surgical aortic valve replacement patients can help identify differences in rehabilitation outcomes between these two groups. However, this kind of study often faces challenges in making direct comparisons because of inherent differences in patient characteristics.

The scope of this narrative review is to provide a broad overview of the current knowledge on CR in TAVI patients while acknowledging the limitations posed by the heterogeneity of existing studies. The considerable variability in sample populations, study designs, CR initiation timelines, and program durations makes direct comparisons challenging. However, by synthesizing the available evidence, this review aims to highlight key findings and trends that may guide future research and clinical practice. The interval between TAVI and the start of CR ranges from as early as the second day post-procedure to over five months. Similarly, the duration of CR programs differs substantially, from just under two weeks to as long as three months. Table 3 summarizes these sources of variability among the studies.

The studies on CR in patients undergoing TAVI measure a wide array of parameters to assess the effectiveness of CR interventions. These parameters help in evaluating not only the physiological changes but also the psychological, functional, and quality-of-life outcomes associated with CR. Table 4 provides a list of the parameters evaluated in the studies and the corresponding tools for measurement.

#### 5.2.1. Functional Capacity

Most of the observational studies published so far in the literature have analyzed the impact of CR on functional capacity, measured either by the 6 min walk test (6MWT) or by maximal oxygen consumption (VO_2_max) reported in cardiopulmonary exercise testing.

6MWT serves as a submaximal exercise test to measure aerobic capacity and endurance. It serves as a practical measure to evaluate the recovery trajectory and guide follow-up care. It has the advantage of being simple, safe, and cost-effective.

A meta-analysis of eleven studies highlighted that exercise-based CR significantly enhances 6MWT performance in TAVI patients, irrespective of the duration of CR intervention [38]. Three studies comparing exercise-based CR with usual care demonstrated significantly greater improvements in 6MWT performance among patients participating in CR. Similar improvements were observed for both short-duration programs (≤3 weeks) and those extending beyond 3 weeks. Another meta-analysis comparing 6 min walking distance before and after CR in TAVI patients also demonstrated a significant statistical improvement [37].

Another recent meta-analysis of 12 observational studies involving 2365 participants confirmed that CR significantly improves 6MWD (6 min walk distance) in patients following TAVI, regardless of program duration (<1 month vs. >1 month) or session frequency (>6 sessions/week vs. <6 sessions/week) [39]. While slightly greater improvements were noted in the longer programs, both high-frequency and moderate-frequency CR sessions yielded comparable benefits in 6MWD. These findings suggest that CR regimens can be effectively tailored to individual patient needs without compromising outcomes.

In contrast, a randomized pilot trial evaluating the effects of an 8-week supervised exercise program, which assigned participants to either a training group or a control group without structured exercise, found no significant difference in the change over time in 6MWT performance between the groups [46]. However, the training group demonstrated significant improvements in other key parameters, including VO_2_max, VO_2_ at anaerobic threshold, CPET duration, and muscle strength.

CPET is an essential component in evaluating and tailoring CR programs, particularly for patients recovering from interventions like transcatheter aortic valve implantation [54]. CPET provides the precise physiological data necessary to assess the effectiveness of rehabilitation and guide program adjustments. However, there are limited data on the impact of CR on CPET parameters compared with more commonly studied outcomes like 6MWD. In addition to the lower availability of CPET, it is a more demanding test, and studies showed that it was tolerated by patients with lower disability and higher exercise capacity [55]. In contrast, studies involving patients with significant disability or reduced exercise capacity often report difficulty tolerating even less strenuous evaluations such as the 6MWD [55]. Thus, due to a scarcity of data, no available meta-analysis exists on the impact of CR on cardiopulmonary function peak VO_2_, metabolic equivalent of task, anaerobic threshold, or respiratory exchange rate.

The randomized pilot trial by Pressler et al. demonstrated the training group significantly improved on the primary endpoint, peak VO_2_. The difference in the change in VO_2_max across groups was 3.7 mL/min/kg (95% CI, 1.1–6.3; *p* = 0.007) [46]. Benefits were observed regarding VO_2_ at the anaerobic threshold (VO_2_AT), with a net gain of 3.2 mL/min/kg (95% CI, 1.6–4.9; *p* < 0.001), and for CPET time, showing an increase of 49 s (95% CI, 6–91; *p* = 0.025). Additionally, all five examined muscle groups showed significant improvements in strength (*p* < 0.010 for all).

A more recent randomized controlled study examined the impact of moderate-intensity continuous training on improving cardiopulmonary function in TAVI patients [40]. After 3 months, the MICT group demonstrated a greater increase in peak VO_2_ compared with the control group, with a mean difference of 1.63 mL/kg/min (95% CI 0.58–2.67, *p* = 0.003). Additionally, the MICT group showed a significantly greater 6MWT, with a change of 21.55 m (95% CI 0.38–42.71, *p* = 0.046).

#### 5.2.2. Frailty and Functional Independence

Frailty is a key factor in determining whether a patient with severe aortic stenosis (AS) should undergo the TAVI procedure rather than surgery [1]. Frailty syndrome is characterized by increased vulnerability to stress due to the decline of multiple physiological systems [56]. It has prognostic value, as it is independently associated with higher mortality and a greater incidence of heart failure hospitalization [57]. The recent recommendation by the European Association of Preventive Cardiology is routine frailty assessment before starting a rehabilitation program, especially in elderly patients (>75 years) [1]. This recommendation is supported by evidence showing that a modified Fried frailty score is independently associated with 1-year mortality in patients following valve replacement [58]. However, frail patients often have limited access to rehabilitation programs, which is why it is essential to assess and overcome barriers to participation in order to increase engagement in these programs [54,59].

There are multiple measurement tools that involve determining cognitive function, nutritional status, functional independence, and geriatric assessment [60,61,62]. The Barthel index (BI) is widely used for assessing frailty and measuring functional independence in activities of daily living (ADLs) [63]. It evaluates a person’s ability to perform basic self-care tasks and mobility activities, providing an indication of their level of dependence on assistance.

A recent meta-analysis reviewed six studies on the impact of CR on BI scores in TAVI patients [39]. The pooled results revealed a significant improvement in BI scores post-CR, with a standardized mean difference (SMD) of 0.83 (95% CI 0.61–1.06; I^2^ = 60.8%). Subgroup analyses based on the duration and frequency of cardiac rehabilitation confirmed that both short-term (less than 1 month) and long-term (more than 1 month) CR programs provided significant benefits. Furthermore, both programs with more than 6 sessions per week as well as those with fewer than 6 sessions per week resulted in notable improvements in BI scores. These findings highlight the effectiveness of CR in enhancing functional independence, regardless of the program’s duration or frequency.

Eichler et al. designed a study that included 136 post-TAVI patients in a rehabilitation program and analyzed its impact on frailty, defined by an index proposed by another author, Schoenenberger [47]. The index includes geriatric questionnaires, MMSE (Mini Mental State Examination) and MNA (Mini Nutritional Assessment), Activities of Daily Living (ADL), Instrumental Activities of Daily Living (IADL); the TUG (Timed Up and Go) test, and a subjective mobility score (probability of walking 200 m or climbing a flight of stairs). The result was positive, as there was a statistically significant decrease of 0.4 points in the frailty index. Compared with pre-rehabilitation measurements, when 47.4% of patients were considered frail according to the index, post-rehabilitation, only 33.3% of the TAVI patients were classified as frail.

A recent meta-analysis evaluated functional independence by comparing BI scores before and after CR programs [39]. Based on data from 4 studies involving 318 participants, the analysis revealed a significant improvement in BI associated with CR (SMD 0.73, 95% CI 0.57–0.89, *p* < 0.01, I^2^ = 42%). Similarly, a study by Kleczynski et al. demonstrated an improved Katz index of ADL at 30 days and 6 months after the CR program [44]. However, the benefit failed to be sustained at 12 months.

#### 5.2.3. Muscular Performance

Including post-TAVI patients in CR programs has been shown to improve muscular strength [46]. Parameters such as handgrip strength, sit-to-stand tests, and one-repetition maximum tests for leg press are commonly used to assess muscular strength. A randomized prospective pilot trial showed significant improvements in both upper and lower body strength as assessed through one-repetition maximum tests for bench press, rowing, pulldown, and shoulder press, favoring the CR program over usual care [46]. Similarly, a randomized controlled trial reported better handgrip strength, with results sustained for 6 and 12 months [44].

However, the improvement in muscular strength was not sustained at 24 ± 6 months [49]. This conclusion was supported by a follow-up analysis of the SPORT pilot trial, which showed that the benefits gained after eight weeks of combined endurance and resistance exercise, when compared with usual care with no training, were not maintained in the long term.

#### 5.2.4. Quality of Life (QoL)

An important objective of post-TAVI cardiac rehabilitation is to enhance the quality of life, which has been measured in studies using various validated questionnaires. These include the Short Form-12 (SF-12) and Short Form-36, which assess physical and mental health, as well as the Kansas City Cardiomyopathy Questionnaire and the Euro-QoL Visual Analog Scale.

The data regarding the impact of cardiac rehabilitation on QoL in TAVI patients are contradictory.

A prospective observational single-arm study by Zanettini et al. found significant improvements in quality of life at discharge for frail TAVI patients, including those with severe functional impairments and high clinical risks [53]. The rehabilitation interventions, tailored to each patient’s needs, resulted in notable improvements in both physical and mental health, highlighting the potential of personalized rehabilitation in high-risk populations.

The persistence of improvements in quality of life after CR failed to be sustained [44,46,49]. Kleczynski et al.’s study demonstrated significant improvements in QoL after CR at 30 days and 6 months, but no such benefits were observed at 12 months [44]. Similarly, a study by Pressler et al. found significant improvements in the aspect of quality of life related to physical function and symptom burden after 8 weeks of CR, but a follow-up study at 24 ± 6 months revealed no differences between CR and usual care, suggesting that the benefits may not be long-lasting [46,49].

In contrast, a recent randomized controlled trial by Hu et al. showed that moderate-intensity continuous training has no impact on QoL, as measured by the SF-12 questionnaire [40]. This indicates that the effects of CR on QoL may vary depending on the specific rehabilitation protocols used.

The mixed findings highlight the need for further research to better understand the impact of CR on QoL in TAVI patients in order to develop adjusted protocols.

#### 5.2.5. Anxiety and Depression

CR also addresses psychological outcomes, but the data regarding the impact are limited and contradictory. For instance, a study by Zanettini et al. found significant reductions in anxiety and depression levels post-TAVI rehabilitation, as assessed by the Hospital Anxiety and Depression Scale (HADS) [53]. These findings suggest that CR, by combining physical activity and psychological support, may have a role in alleviating anxiety and depression, improving overall well-being in TAVI patients. A recent meta-analysis by Hosseinpour et al., comparing pre- and post-training HADS among 4 studies including 318 participants, showed no difference in the HADS-A (anxiety) score but a significant reduction in the HADS-D (depression) score following the exercise training program [38].

On the other hand, some studies report no significant effect on anxiety and depression despite improvements in physical outcomes [44,50].

Further long-term follow-up studies are needed to explore the potential for sustained improvements in mental health outcomes post-CR.

#### 5.2.6. Echocardiography and Laboratory Parameters

CR has no significant impact on left ventricular ejection fraction or renal function [45,46,49]. CR is safe, with no prosthesis dysfunction reported [53]. However, there is a scarcity of studies in this domain, highlighting a significant gap in the literature. This underscores the need for further research to provide more comprehensive insights and evidence-based recommendations.

#### 5.2.7. Mortality

The impact of CR on outcomes such as mortality and hospitalization in post-TAVI patients remains insufficiently studied. Two studies indicate that CR may lower mortality rates, particularly important in high-risk post-TAVI patients [45,49]. However, these benefits are usually more commonly observed during extended follow-up periods. A notable study by Butter et al. examined this association in a cohort of 1017 patients offered rehabilitation [45]. Among them, 36% declined participation, while 41.8% underwent cardiac rehabilitation and 21.2% participated in geriatric rehabilitation. At six months, survival rates were significantly higher in the rehabilitation group compared with non-participants (95% vs. 89.8%, *p* = 0.003). Multivariate analysis confirmed rehabilitation as an independent predictor of reduced six-month mortality, driven largely by reduced non-cardiac mortality. Interestingly, cardiac rehabilitation demonstrated greater survival benefits than geriatric rehabilitation. However, rehospitalization rates did not differ between the groups nor when compared with those who declined rehabilitation. The observational design of Butter et al.’s study suggests potential biases, as those refusing rehabilitation were more likely to face depression, socio-economic challenges, or physical inactivity—factors correlated with poorer survival.

Complementing this, the SPORT TAVI trial, a pilot randomized study, suggested a trend toward improved survival at 24 months in TAVI patients undergoing cardiac rehabilitation, though the results were not statistically significant due to the limited sample size [49].

These findings highlight the potential of CR to enhance survival, particularly through reductions in non-cardiac mortality, while emphasizing the need for larger randomized studies to confirm these benefits and refine rehabilitation strategies for TAVI patients.

### 5.3. Limitations

One of the most important limitations is the scarcity of evidence-based data. Few studies were found that address the issue of cardiac rehabilitation in post-TAVI patients. As a consequence, we have included studies despite their heterogeneity in order to provide context and a comprehensive perspective, which is essential for a narrative review. However, the significant heterogeneity in terms of design, patient population, and measured outcomes makes it difficult to draw definitive conclusions about the impact of cardiac rehabilitation in TAVI patients. The study designs range from retrospective observational studies to randomized controlled trials, with considerable variability in sample size, patient characteristics, and rehabilitation protocols. The majority of the studies are observational and lack robust control groups, making it difficult to establish causality. Some studies focus exclusively on TAVI patients, while others include comparisons with SAVR patients, further complicating the interpretation of results. Additionally, there is a notable lack of long-term follow-up, as many studies assess outcomes at only one to three months, which limits the ability to evaluate the sustained benefits of CR over time. Another major limitation is the predominant focus on functional and quality-of-life measures, such as the six-minute walk test, frailty indices, and self-reported questionnaires, rather than hard clinical endpoints like cardiovascular mortality, hospital readmission, or major adverse cardiovascular events. Although some studies, such as that of Butter et al. [45], suggest that CR may reduce mortality, the evidence remains insufficient due to methodological differences and the lack of large-scale, randomized trials with extended follow-up periods.

There is a wide variability regarding the duration of the intervention, the type of exercise recommended, and the frequency and intensity of sessions. Regarding exercise regimens and intensity, there was some variability in exercise types across the studies, including walking, cycling, resistance training, and endurance exercises. Most studies prescribed moderate-intensity exercise, and some tailored the intensity based on patient assessment [40,43]. Cardiac rehabilitation program durations ranged from 3 weeks to 3 months, with the most common frequency being 2–3 times per week, though several studies utilized daily regimens for residential CR [47,48]. These factors underline the need for larger, more rigorous randomized controlled trials to establish standardized CR programs tailored to TAVI patients, with a focus on refining exercise protocols, intensity levels, and follow-up periods to maximize therapeutic outcomes.

Evidence on how CR impacts metabolic responses post-TAVI is limited. Further investigations are required to evaluate the specific metabolic effects of CR, particularly its influence on parameters like glomerular filtration rate, lipid profiles, glucose metabolism, and overall metabolic health. Understanding these responses could provide valuable insights into optimizing CR protocols for TAVI patients, especially those with preexisting metabolic or renal impairments.

Given these limitations, there is a pressing need for well-designed, multicenter, randomized controlled trials with long-term follow-up to definitively determine whether CR has a causal impact on major clinical outcomes in TAVI patients. Future research should aim to standardize CR protocols, incorporate objective biomarkers, and assess long-term cardiovascular morbidity and mortality.

## 6. Conclusions

In conclusion, cardiac rehabilitation plays an important role in post-TAVI care, as it is associated with improvements in exercise capacity, survival, muscular strength, and functional independence. It shows a promising trend in augmenting quality of life and alleviating the symptoms of anxiety and depression, although the evidence is not yet fully conclusive. Notably, CR has been shown to have a good safety profile, thus supporting its inclusion as a key component of therapeutic management. Follow-up periods of up to 18–24 months have indicated that the benefits of CR are sustained in the medium term, particularly in terms of improving functional status. The variability in exercise protocols across studies highlights the need for a more standardized approach. Additionally, while CR has shown benefits in a broad panel of health outcomes, research is scarce regarding its metabolic effects. Addressing these gaps could lead to more personalized and effective CR programs, improving long-term outcomes in this high-risk population. Further multicenter randomized controlled trials with long-term follow-up are needed to provide more conclusive evidence on the role of CR in this patient population.

## Figures and Tables

**Table 1:** Overview of key meta-analyses.

Study/ Year/ Country	No. of Patients	Measurements	Follow-Up	Main Findings
**CR impact in TAVI vs. SAVR patients**				
Ribeiro et al., 2017 [35]	292 TAVI 570 SAVR	Functional capacity (oxygen uptake and/or workload); exercise tolerance (walked distance, exercise time), 6MWT, BI, FIM, HADS, EQ VAS, data on all-cause and cardiovascular mortality, safety outcomes	Not mentioned	Significant and similar improvement in 6MWD and BI in both TAVI and SAVR patients; CR is safe in both groups.
Anayo et al., 2019 [36]	27 TAVI 99 SAVR 129 mixed patients	VO_2_max, 6MWT, SF-12 or SF-36, KCCQ, NYHA class, return to work, adverse effects of CR, costs	2–12 months	In the short term, exercise-based CR enhances exercise capacity in patients who have undergone TAVI or SAVR.
**CR impact in TAVI patients**				
Oz et al., 2023 [37]	Data for 6MWT: 456 TAVI patients Data for BI: 377 TAVI patients	6MWT, BI	Before and after CR; the usual duration of CR was 3 weeks in most studies	CR improves exercise tolerance and functional independence measured by 6MWT and BI. CR is safe in this group.
Hosseinpour et al., 2024 [38]	685 TAVI patients	6MWT, BI, HADS, SF-12	Before and after CR; duration of CR lasted from 2 weeks to 2 months	CR improves 6WMT, Barthel index, SF-12 and mental survey scores, HADS scores.
Li et al., 2023 [39]	253 TAVI patients	6MWT, BI, SF-12, ExCap, MFS, frailty index, FIM score	Before and after CR; duration of CR lasted 16.4 ± 3.9 days; follow-up less than 6 months	CR improves 6MWD and Barthel index irrespective of CR duration/frequency. CR improves QoL, Morse Fall Scale, frailty index. CR improves exercise capacity.

TAVI, transcatheter aortic valve implantation; SAVR, surgical aortic valve replacement; 6WMT, 6 min walk test; 6MWD, 6 min walk distance; BI, Barthel index; FIM, functional independence measure score; HADS, Hospital Anxiety and Depression Scores; EQ VAS, EuroQol visual analog scale; VO_2_max, maximal oxygen uptake; SF-12, 12-item Short-Form Survey questionnaire; SF-36, 36-item Short-Form Survey questionnaire; KCCQ, Kansas City Cardiomyopathy Questionnaire; NYHA class, New York Heart Association classification of heart failure; ExCap, exercise capacity; MFS, Morse Fall Scale.

**Table 2:** Overview of main clinical trials.

Study (Year)/ Country/No. Tavi Patients/ Reference	Type of Study	Measurements	Follow-Up	Main Findings
Hu et al. (2023) China 66 [40]	Prospective randomized controlled trial comparing moderate-intensity continuous training vs. control	VO_2_max, VO_2_ at AT, MET at AT, 6MWT, SF-12, echocardiography, NYHA class, MACE	3 months	Improved cardiopulmonary function Improved physical capacity (Δ peak VO_2_ 1.63 mL/kg/min [95% CI 0.58–2.67, *p* = 0.003]; Δ 6MWD 21.55 m, [95% CI 0.38–42.71, *p* = 0.046]) Lower low-density lipoprotein cholesterol (Δ 0.62 mmol/L [95% CI e1.00 to _0.23, *p* = 0.002])
Xu et al. (2023) China 96 [41]	Double-blinded, sham-controlled randomized clinical trial comparing CR vs. CR plus inspiratory muscle training (IMT)	6MWT; lung function FVC, FEV_1_, FEV_1/FVC,_ MIP, MEP, FIVC; limb muscle force HGS, sit-to-stand test, arm-curl test Functional status mMRC, DASI, PSQI, BI, FS-14, SF-12	Baseline, discharge of CR, 1 month, and 3 months	CR plus IMT enhances exercise endurance, pulmonary ventilation function, and inspiratory muscle strength and shortens the length of hospital stay (11 days vs. 12.5 days, *p* = 0.016). Δ 6MWD 33.52m [95% CI: −64.42 to −2.62, *p* = 0.034]. Effects persistent at 1 month and 3 months.
Yu Z et al. (2021) China 90 [42]	Retrospective observational evaluating CR in TAVI	BI, MMSE, MNA, HADS, frailty by Fried scale, 6MWD, MET	1 month	Cognitive impairment, malnutrition, and frailty significantly decreased by 21%, 40%, and 57%, respectively (*p* = 0.002, *p* < 0.001, *p* < 0.001). Improved 6MWD 218.8 m ± 114.3 m to 291.9 m ± 98.8 (*p* < 0.001); frailty and malnutrition are predictors of 6MWD improvement.
Penati et al. (2021) Italy 46 [43]	Prospective observational evaluating CR in TAVI patients	6MWT, SPPB, BI	Baseline, discharge, 18 months	Significantly improved all evaluated measures; BI from 73.80 ± 23.31 to 90.22 ± 16.53 at discharge (*p* < 0.001); Δ 6MWD from 265.43 m ± 89 to 327.17 m ± 111 at discharge (*p* = 0.043); SPPB from 4.56 ± 2.27 to 7.13 ± 3.08 at discharge (*p* = 0.002).
Kleczynski (2021) Poland 105 [44]	Retrospective cohort comparing CR vs. no CR	Frailty indices: 5MWT, 6MWT, HGS, KI of ADL; QoL: HADS, KCCQ	Baseline, 30 days, 6 months, 12 months	Improved clinical performance and quality of life, but these outcomes declined after one year. A longer time interval following the completion of inpatient CR is associated with a decline in performance.
Butter et al. (2018) Germany 1056 [45]	Longitudinal cohort study, multicenter, comparing rehabilitation vs. no rehabilitation and comparing cardiac rehabilitation vs. geriatric rehabilitation	Mortality at 6 months	6 months	Cardiac rehabilitation reduces mortality (adjusted OR: 0.31; 95% CI 0.14–0.71, *p* = 0.006).
Pressler et al. (2018) Germany 17 [46]	Randomized pilot study CR vs. no CR in TAVI	VO_2_ peak, VO_2_AT; muscular strength, 6MWD; QoL, KCCQ, SF-12; NYHA class, echocardiography; NT-proBNP, creatinine, GFR	24 ± 6 months	Significant long-term improvements in submaximal exercise performance (Δ VO_2_AT 2.7 mL/min/kg [95% CI 0.8–4.6, *p* < 0.008]) were maintained. Not the case for VO_2_peak, muscular strength, or quality of life.
Eichler et al. (2016) Germany 136 [47]	Prospective cohort multicenter study evaluating CR in TAVI	6MWT, SF-12, HADS, frailty index that includes MMSE, MNA, ADL, IADL, TUG, subjective mobility disability	Baseline, discharge	Improved 6MWD and maximum exercise capacity from 56.3 ± 65.3 m (*p* < 0.001) and 8.0 ± 14.9 watts (*p* < 0.001), reduced proportion of frail patients by 9%. Improved quality of life (Δ SF-12 physical 2.5 ± 8.7, *p* < 0.001, mental 3.4 ± 10.2, *p* < 0.003); reduced anxiety (HADS from 5.2 ± 4.0 to 4.0 ± 3.6, *p* < 0.001).
Tarro Genta et al. (2017) Italy 65 [48]	Prospective observational, CR after TAVI compared with CR after SAVR	6MWT, CIRS-CI BI, MFS, echocardiography	3 weeks	Improved 6MWT from 162 m ± 92 to 240 m ± 92; *p* < 0.001; improved BI from 67 ± 24 to 85 ± 17, *p* < 0.001; MFS difference admission vs. discharge: 5.8 ± 20, *p* < 0.02; CR is safe.
Pressler et al. (2016) Germany 27 [49]	Randomized pilot trial comparing CR vs. no CR in TAVI	VO_2_ peak, VO_2_AT, RER, Ve/VCO2, max HR, 1-RM on 5 different machines, 6MWT, echocardiography; QoL KCCQ, SF-12; NT-proBNP creatinine, GFR	Baseline, after CR	Improvements in exercise capacity VO_2_ max (Δ 3.7 mL/min per kg [95% CI, 1.1–6.3; *p* = 0.007]) but not 6MWD; improvement in quality of life (KCCQ physical limitation Δ 19.2 [95% CI, 4.1–34.2; *p* = 0.015]) and in muscular strength; CR is safe.
Voller (2015) Germany 76 [50]	Observational study and propensity score analysis TAVI and SAVR after CR	Functional evaluation 6MWT and exercise capacity on bicycle exercise test; emotional evaluation, HADS	3 weeks	Improved exercise capacity, increased by 19.84% (95% CI: 20.59 to 36.15, *p* < 0.05) and 6MWT increased by 28.13% (95% CI: 20.59 to 36.15, *p* < 0.001). No effect on anxiety and depression.
Fauchere (2014) Switzerland 34 [51]	Retrospective observational study comparing CR in TAVR vs. CR in SAVR	FIM score, HADS, 6MWT	Admission, discharge	Improvement in 6MWT (from 147.5 m ± 101.7 to 231.7 ± 132.7, *p* < 0.001), FIM (from 95.8 ± 10.2 to 106.8 ± 9.9, *p* < 0.001) in TAVI; improvement observed in both groups.
Russo et al. (2014) Italy 78 [52]	Prospective observational study comparing CR in TAVI vs. CR in SAVR	6MWT, CPET, echocardiography, BI	Baseline, discharge	CR is feasible, safe, and effective in octogenarians after TAV; enhances functional capacity, Δ 6MWT 60.4 ± 46.4 m, *p* < 0.001.
Zanettini et al. (2014) Italy 60 [53]	Prospective observational single-arm evaluating CR in TAVI	Echocardiographic parameters, 6MWT, modified BI, MMSE, GDS, EQ-5D, EQ-VAS	T1 6–12 month T2 18–24 months	Improved functional status: Δ 6MWD from 210 m ± 87 to 275 m ± 97, *p* < 0.001, Δ BI from 84 ± 21 to 95 ± 10, *p* < 0.001; quality of life: Δ EQ-VAS from 54 ± 14 to 75 ± 11, *p* < 0.001. Results stable in mid-term follow-up.

TAVI, transcatheter aortic valve implantation; SAVR, surgical aortic valve replacement; CIRS-CI, Cumulative Illness Rating Scale; BI, Barthel index; MFS, Morse Fall Scale; 6MWT, 6 min walk test; 6MWD, 6 min walk distance; VO_2_max, maximal oxygen uptake; VO_2_AT, oxygen uptake at anaerobic threshold; MET at AT, metabolic equivalent at anaerobic threshold; SF-12, 12-item Short-Form Survey questionnaire; NYHA class, New York Heart Association classification of heart failure; MACE, major adverse cardiovascular events; FVC, forced vital capacity; FEV_1_, forced expiratory volume in one second; MIP, maximal inspiratory pressure; MEP, maximal expiratory pressure; FIVC, forced inspiratory vital capacity; HGS, handgrip strength; mMRC, modified Medical Research Council scale; DASI, Duke Activity Status Index; PSQI, Pittsburgh Sleep Quality Index; FS-14, Fatigue Scale 14; NT-proBNP, N-terminal pro-B-type natriuretic peptide; MMSE, Mini Mental State Examination; MNA, Mini Nutritional Assessment; HADS, Hospital Anxiety and Depression Scale; peak VO_2_, maximum oxygen uptake; MET, metabolic equivalent; SPPB, Short Physical Performance Battery; 5MWT, 5 m walk time; KI of ADL, Katz index of Independence of Activities in Daily Living; KCCQ, Kansas City Cardiomyopathy Questionnaire; GFR, glomerular filtration rate; IADL, Instrumental Activities of Daily Living; TUG, Timed Up and Go; RER, respiratory exchange rate; Ve/VCO2, minute ventilation/carbon dioxide production; max HR, maximum heart rate; 1-RM, one repetition maximum; CPET, cardiopulmonary exercise testing; GDS, the Geriatric Depression Scale; EQ-5D, the EuroQol Questionnaire in 5 domains; EQ-VAS, the EQ visual analog scale.

**Table 3:** Interval time between TAVI and CR beginning and the duration of CR among the studies.

Study (Year)/ Country/No. Tavi Patients [Reference]	Interval Time Between TAVI and CR Beginning	Duration of CR
Hu et al. (2023) China, 66 [40]	157 days (35.0–200.25 days)	3 months
Xu et al. (2023) China, 96 [41]	CR program began the second day following the TAVI procedure.	12.5 days [10.00–14.00 days]
Yu Z et al. (2021) China, 90 [42]	Before discharge after TAVI facilitation	1 month
Penati et al. (2021) Italy, 46 [43]	Not mentioned	Average of 3 weeks of rehabilitation; exact number of days not mentioned
Kleczynski (2021) Poland, 105 [44]	Immediately after discharge from TAVI facilitation; exact days of the procedure not mentioned.	14-night stay
Butter et al. (2018) Germany, 1056 [45]	7.0 days; interquartile range: 6.0, 9.0 days	3 weeks
Pressler et al. (2018) Germany, 17 [46]	14 days	8 weeks
Eichler et al. (2016) Germany, 136 [47]	17.7 ± 9.9 days	19.4 ± 3.1 days
Tarro Genta et al. (2017) Italy, 65 [48]	11 ± 6 days	25 ± 11 days
Pressler et al. (2016) Germany, 27 [49]	83 ± 34 days	8 weeks
Voller (2015) Germany, 76 [50]	24.05 ± 15.82 days	19.17 ± 4.54 days
Fauchere (2014) Switzerland, 34 [51]	Not mentioned	19.2 ± 6.4 days
Russo et al. (2014) Italy, 78 [52]	13.7 ± 11.7 days	16.6 ± 4.7 days
Zanettini (2014) Italy, 60 [53]	10.6 ± 3.4 days	18.3 ± 5.6 days

**Table 4:** List of main parameters evaluated in studies.

Parameters	Tools for Measurement	References
Functional capacity	6MWT, Peak VO_2_, VO_2_ at AT, metabolic equivalent, MET at AT, bicycle exercise test	[35,36,37,38,40,41,43,44,47,48,49,52,53]
Frailty and functional independence	KI of ADL, mMRC, DASI, BI, FS-14, 5MWT	[35,36,37,38,41,42,43,48,52,53]
Muscular strength	HGS, sit-to-stand test, arm-curl test	[41,42,44,46]
Quality of life	KCCQ, EQ-VAS, SF-12, SF-36, EQ-5D, PSQI	[35,36,42,44,46,47,49,51,53]
Depression and anxiety	HADS	[35,36,38,40,42,44,45,47,48,49,50,51]
Lung function	FVC, FEV_1_, FEV_1/FVC_, MIP, MEP, FIVC	[41]
Multimorbidity	CIRS-CI	[48]

6MWT, 6 min walk test; Peak VO_2_, maximal oxygen uptake; VO_2_ at AT, oxygen uptake at anaerobic threshold; MET, metabolic equivalent; MET at AT, metabolic equivalent at anaerobic threshold; KI of ADL, Katz index of Independence of Activities in Daily Living; mMRC, modified Medical Research Council scale; DASI, Duke Activity Status Index; BI, Barthel index; FS-14, Fatigue Scale 14; 5MWT, 5 m walk time; HGS, hand grip strength; KCCQ, Kansas City Cardiomyopathy Questionnaire; EQ-VAS, EuroQol visual analog scale; SF-12, 12-item Short-Form Survey questionnaire; SF-36, 36-item Short-Form Survey questionnaire; EQ-5D, the EuroQol Questionnaire in 5 domains; PSQI, Pittsburgh Sleep Quality Index; HADS, Hospital Anxiety and Depression Scale; FVC, forced vital capacity; FEV_1_, forced expiratory volume in one second; MIP, maximal inspiratory pressure; MEP, maximal expiratory pressure; FIVC, forced inspiratory vital capacity; CIRS-CI, Cumulative Illness Rating Scale.

## Abbreviations

The following abbreviations are used in this manuscript:

ADL	Activities of Daily Living
AS	Aortic stenosis
BI	Barthel index
CPET	Cardiopulmonary exercise testing
CR	Cardiac rehabilitation
HADS	Hospital Anxiety and Depression Scale
HRV	Heart rate variability
MICT	Moderate-intensity continuous training
QoL	Quality of life
SF-12	Short Form-12
TAVI	Transcatheter aortic valve implantation
VO_2_max	Maximal oxygen uptake
WG	Working group
6MWD	6 min walk distance
6MWT	6 min walk test
